# 1-Phenyl-2-(1*H*-1,2,4-triazol-1-yl)ethanone

**DOI:** 10.1107/S1600536808023258

**Published:** 2008-07-26

**Authors:** Özden Özel Güven, Hakan Tahtacı, Simon J. Coles, Tuncer Hökelek

**Affiliations:** a Zonguldak Karaelmas University, Department of Chemistry, 67100 Zonguldak, Turkey; b Southampton University, Department of Chemistry, Southampton SO17 1BJ, England; c Hacettepe University, Department of Physics, 06800 Beytepe, Ankara, Turkey

## Abstract

In the mol­ecule of the title compound, C_10_H_9_N_3_O, the triazole and phenyl rings are nearly perpendicular to each other, with a dihedral angle of 88.72 (4)°. In the crystal structure, inter­molecular C—H⋯O and C—H⋯N hydrogen bonds link the mol­ecules. There are C—H⋯π contacts between the 1,2,4-triazole rings, and between the phenyl and 1,2,4-triazole rings, and there is a weak π–π contact between the 1,2,4-triazole and phenyl rings [centroid-to-centroid distance = 4.547 (1) Å].

## Related literature

For general background, see: Holla *et al.* (1996 ▶); Sengupta *et al.* (1978 ▶); Paulvannan *et al.* (2001 ▶); Sui *et al.* (1998 ▶); Bodey (1992 ▶). For related literature, see: Caira *et al.* (2004 ▶); Peeters *et al.* (1996 ▶); Özel Güven, Tahtacı *et al.* (2008 ▶); Özel Güven, Erdoğan *et al.* (2008 ▶). For synthesis, see: Liu *et al.* (2006 ▶).
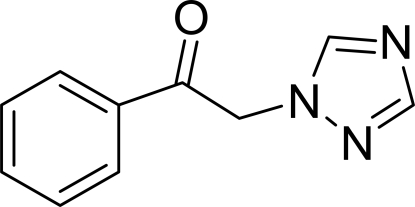


## Experimental

### 

#### Crystal data

C_10_H_9_N_3_O
*M* *_r_* = 187.20
Orthorhombic, 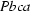

*a* = 9.3129 (2) Å
*b* = 8.11660 (10) Å
*c* = 24.0475 (4) Å
*V* = 1817.73 (5) Å^3^
*Z* = 8
Mo *K*α radiation
μ = 0.09 mm^−1^
*T* = 120 (2) K
0.35 × 0.2 × 0.2 mm

#### Data collection

Bruker Nonius KappaCCD diffractometer
Absorption correction: multi-scan (*SADABS*; Sheldrick, 2007 ▶) *T*_min_ = 0.968, *T*_max_ = 0.972
16799 measured reflections
2077 independent reflections
1736 reflections with *I* > 2σ(*I*)
*R*_int_ = 0.052

#### Refinement

*R*[*F* ^2^ > 2σ(*F* ^2^)] = 0.060
*wR*(*F* ^2^) = 0.145
*S* = 1.17
2077 reflections
164 parameters
All H-atom parameters refined
Δρ_max_ = 0.56 e Å^−3^
Δρ_min_ = −0.55 e Å^−3^

### 

Data collection: *COLLECT* (Nonius, 1998 ▶); cell refinement: *DENZO* (Otwinowski & Minor, 1997 ▶) and *COLLECT*; data reduction: *DENZO* and *COLLECT*; program(s) used to solve structure: *SHELXS97* (Sheldrick, 2008 ▶); program(s) used to refine structure: *SHELXL97* (Sheldrick, 2008 ▶); molecular graphics: *ORTEP-3 for Windows* (Farrugia, 1997 ▶) and *PLATON* (Spek, 2003 ▶); software used to prepare material for publication: *WinGX* (Farrugia, 1999 ▶) and *PLATON*.

## Supplementary Material

Crystal structure: contains datablocks I, global. DOI: 10.1107/S1600536808023258/xu2439sup1.cif

Structure factors: contains datablocks I. DOI: 10.1107/S1600536808023258/xu2439Isup2.hkl

Additional supplementary materials:  crystallographic information; 3D view; checkCIF report

## Figures and Tables

**Table 1:** Hydrogen-bond geometry (Å, °) *Cg*1 and *Cg*2 are the centroids of the rings N1–N3/C1/C2 and C5–C10, respectively.

*D*—H⋯*A*	*D*—H	H⋯*A*	*D*⋯*A*	*D*—H⋯*A*
C2—H2⋯O^i^	0.970 (16)	2.449 (15)	3.2595 (17)	140.9 (12)
C3—H32⋯O^i^	0.973 (16)	2.489 (16)	3.2601 (17)	136.1 (13)
C8—H8⋯N3^ii^	0.97 (2)	2.61 (2)	3.5405 (19)	160.2 (14)
C1—H1⋯*Cg*2^iii^	1.001 (17)	2.840 (18)	3.620 (2)	135.20 (13)
C2—H2⋯*Cg*1^iv^	0.972 (17)	3.013 (16)	3.829 (2)	142.42 (12)

Symmetry codes: (i) 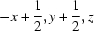; (ii) 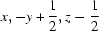; (iii) 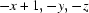; (iv) 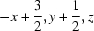.

**Fig. 1.**
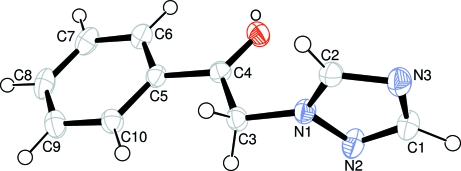
The molecular structure of the title molecule with the atom-numbering scheme. Displacement ellipsoids are drawn at the 50% probability level.

**Fig. 2.**
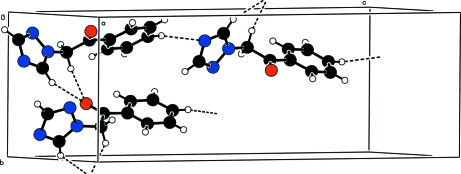
A packing diagram of (I). Hydrogen bonds are shown as dashed lines.

## supplementary crystallographic information

### Comment

In recent years, among antifungal agents, azole derivatives still have an important place in the class of systemic antifungal drugs. 1,2,4-Triazoles are biologically interesting molecules and their chemistry is receiving considerable attention, due to antihypertensive, antifungal and antibacterial properties (Holla *et al.*, 1996; Sengupta *et al.*, 1978; Paulvannan *et al.*, 2001; Sui *et al.*, 1998). The azole antifungals possessing a triazole ring such as fluconazole (Caira *et al.*, 2004) and itraconazole (Peeters *et al.*, 1996) inhibit the synthesis of sterols in fungi by inhibiting cytochrome P-450-dependent 14α-lanosterol demethylase (P-450_14DM_) and prevent cytochrome P-450 activity (Bodey, 1992). Recently, we reported the crystal structures of 1,2,4-triazole substituted alcohol (Özel Güven, Tahtacı *et al.*, 2008) and benzimidazole substituted ketone (Özel Güven, Erdoğan *et al.*, 2008). We report herein the crystal structure of the 1,2,4-triazole substituted ketone, (I).

In (I), the bond lengths and angles are generally within normal ranges (Fig. 1). The 1,2,4-triazole and benzene rings, A (N1–N3/C1/C2) and B (C5–C10), are, of course, planar and nearly perpendicular to each other with a dihedral angle of A/B = 88.72 (4)°. Atoms C3 and C4 are -0.028 (2) Å and -0.054 (1) Å away from the ring planes of A and B, respectively. The N1—C3—C4 [112.73 (11)°], C3—C4—C5 [116.93 (11)°], O—C4—C3 [120.73 (12)°] and O—C4—C5 [122.34 (12)°] bond angles are highly different from the corresponding values [111.53 (10)°, 109.94 (10)°, 109.53 (11)° and 110.01 (10)°, respectively] in 1-phenyl-2-(1*H*-1,2,4-triazol-1-yl)ethanol, (II) (Özel Güven, Tahtacı *et al.*, 2008). In ring A, the nearly equivalent N1—N2—C1 [101.81 (11)°] and C1—N3—C2 [102.23 (11)°] bond angles are narrowed, while highly different N3—C2—N1 [110.53 (13)°] and N3—C1—N2 [115.39 (12)°] bond angles are enlarged, as in (II).

In the crystal structure, intermolecular C—H···O and C—H···N hydrogen bonds (Table 1) link the molecules (Fig. 2), in which they seem to be effective in the stabilization of the structure. The C—H···π contacts (Table 1) between the 1,2,4-triazole and the benzene rings and the 1,2,4-triazole rings and a π—π contact between the 1,2,4-triazole and benzene ring systems *Cg*2···*Cg*1^i^ [symmetry code: (i) 1 - *x*, -*y*, -*z*, where *Cg*1 and *Cg*2 are centroids of the rings (N1–N3/C1/C2) and (C5–C10), respectively] further stabilize the structure, with centroid–centroid distance of 4.547 (1) Å.

### Experimental

The title compound, (I), was synthesized by the reaction of 1*H*-1,2,4-triazole with 2-bromo-1-phenylethanone (Liu *et al.*, 2006). To a vigorous stirred suspension of 1*H*-1,2,4-triazole (1105 mg, 16 mmol) and 2-bromo-1-phenylethanone (1990 mg, 10 mmol) in acetone (6 ml) was added triethylamine (2.2 ml) dropwise over a period of 1 h below 273 K, and the reaction mixture was stirred for another 30 min at room temperature. Then the mixture was filtered to remove triethylamine hydrobromide salt, the precipitate was washed with acetone, and the filtrate was evaporated under reduced pressure. The residue was dissolved in chloroform, and washed with water. After evaporation of chloroform, the yellow solid was obtained and crystallized from iso-propanol to obtain the title compound as colorless crystals (yield; 937 mg, 50%).

### Refinement

H atoms were located in difference syntheses and refined isotropically [C—H = 0.954 (18)–1.007 (17) Å, *U*_iso_(H) = 0.030 (4)–0.046 (5) Å^2^].

### Crystal data

**Table:**

C_10_H_9_N_3_O	*F*_000_ = 784
*M**_r_* = 187.20	*D*_x_ = 1.368 Mg m^−^^3^
Orthorhombic, *P**b**c**a*	Mo *K*α radiation λ = 0.71073 Å
Hall symbol: -P 2ac 2ab	Cell parameters from 2395 reflections
*a* = 9.3129 (2) Å	θ = 2.9–27.5º
*b* = 8.11660 (10) Å	µ = 0.09 mm^−^^1^
*c* = 24.0475 (4) Å	*T* = 120 (2) K
*V* = 1817.73 (5) Å^3^	Shard, colourless
*Z* = 8	0.35 × 0.2 × 0.2 mm

### Data collection

**Table:**

Bruker Nonius KappaCCD diffractometer	2077 independent reflections
Radiation source: fine-focus sealed tube	1736 reflections with *I* > 2σ(*I*)
Monochromator: graphite	*R*_int_ = 0.052
Detector resolution: 9.091 pixels mm^-1^	θ_max_ = 27.5º
*T* = 120(2) K	θ_min_ = 3.4º
φ and ω scans	*h* = −12→12
Absorption correction: multi-scan(SADABS; Sheldrick, 2007)	*k* = −10→9
*T*_min_ = 0.968, *T*_max_ = 0.972	*l* = −31→31
16799 measured reflections	

### Refinement

**Table:**

Refinement on *F*^2^	Hydrogen site location: inferred from neighbouring sites
Least-squares matrix: full	All H-atom parameters refined
*R*[*F*^2^ > 2σ(*F*^2^)] = 0.060	*w* = 1/[σ^2^(*F*_o_^2^) + (0.0862*P*)^2^ + 0.1878*P*] where *P* = (*F*_o_^2^ + 2*F*_c_^2^)/3
*wR*(*F*^2^) = 0.145	(Δ/σ)_max_ < 0.001
*S* = 1.17	Δρ_max_ = 0.56 e Å^−^^3^
2077 reflections	Δρ_min_ = −0.55 e Å^−^^3^
164 parameters	Extinction correction: SHELXL97 (Sheldrick, 2008), Fc^*^=kFc[1+0.001xFc^2^λ^3^/sin(2θ)]^-1/4^
Primary atom site location: structure-invariant direct methods	Extinction coefficient: 0.141 (11)
Secondary atom site location: difference Fourier map	

### Special details

**Table:**

Geometry. All e.s.d.'s (except the e.s.d. in the dihedral angle between two l.s. planes) are estimated using the full covariance matrix. The cell e.s.d.'s are taken into account individually in the estimation of e.s.d.'s in distances, angles and torsion angles; correlations between e.s.d.'s in cell parameters are only used when they are defined by crystal symmetry. An approximate (isotropic) treatment of cell e.s.d.'s is used for estimating e.s.d.'s involving l.s. planes.
Refinement. Refinement of *F*^2^ against ALL reflections. The weighted *R*-factor *wR* and goodness of fit *S* are based on *F*^2^, conventional *R*-factors *R* are based on *F*, with *F* set to zero for negative *F*^2^. The threshold expression of *F*^2^ > σ(*F*^2^) is used only for calculating *R*-factors(gt) *etc*. and is not relevant to the choice of reflections for refinement. *R*-factors based on *F*^2^ are statistically about twice as large as those based on *F*, and *R*- factors based on ALL data will be even larger.

### Fractional atomic coordinates and isotropic or equivalent isotropic displacement parameters (Å^2^)

**Table:**

	*x*	*y*	*z*	*U*_iso_*/*U*_eq_	
O	0.27304 (10)	0.10771 (12)	0.46535 (4)	0.0301 (3)	
N1	0.08642 (12)	0.25265 (13)	0.53706 (5)	0.0237 (3)	
N2	0.04051 (14)	0.12395 (15)	0.56849 (5)	0.0306 (4)	
N3	0.15796 (13)	0.31174 (16)	0.62134 (5)	0.0302 (3)	
C1	0.08629 (16)	0.16652 (19)	0.61863 (6)	0.0302 (4)	
H1	0.0645 (19)	0.097 (2)	0.6520 (7)	0.037 (4)*	
C2	0.15570 (14)	0.36160 (18)	0.56884 (6)	0.0256 (3)	
H2	0.1995 (17)	0.460 (2)	0.5535 (6)	0.030 (4)*	
C3	0.05623 (16)	0.25908 (17)	0.47805 (5)	0.0241 (3)	
H31	−0.0374 (19)	0.210 (2)	0.4711 (6)	0.032 (4)*	
H32	0.0550 (18)	0.373 (2)	0.4655 (7)	0.035 (4)*	
C4	0.16946 (13)	0.17185 (16)	0.44337 (5)	0.0229 (3)	
C5	0.14815 (14)	0.17126 (16)	0.38202 (5)	0.0231 (3)	
C6	0.25102 (16)	0.09475 (17)	0.34846 (6)	0.0284 (4)	
H6	0.3339 (19)	0.048 (2)	0.3658 (7)	0.038 (5)*	
C7	0.23147 (18)	0.08608 (18)	0.29144 (6)	0.0333 (4)	
H7	0.3021 (18)	0.033 (2)	0.2690 (7)	0.035 (4)*	
C8	0.11013 (19)	0.15544 (19)	0.26698 (6)	0.0352 (4)	
H8	0.0997 (19)	0.151 (2)	0.2269 (9)	0.046 (5)*	
C9	0.00879 (18)	0.23382 (18)	0.29988 (6)	0.0320 (4)	
H9	−0.0794 (18)	0.281 (2)	0.2837 (7)	0.036 (4)*	
C10	0.02666 (15)	0.24132 (16)	0.35721 (6)	0.0263 (4)	
H10	−0.0483 (18)	0.296 (2)	0.3812 (6)	0.031 (4)*	

### Atomic displacement parameters (Å^2^)

**Table:**

	*U*^11^	*U*^22^	*U*^33^	*U*^12^	*U*^13^	*U*^23^
O	0.0267 (5)	0.0372 (6)	0.0263 (5)	0.0052 (4)	−0.0022 (4)	0.0031 (4)
N1	0.0264 (6)	0.0252 (6)	0.0194 (6)	−0.0012 (4)	0.0005 (4)	0.0005 (4)
N2	0.0414 (7)	0.0283 (6)	0.0221 (6)	−0.0054 (5)	0.0016 (5)	0.0028 (5)
N3	0.0340 (7)	0.0349 (7)	0.0216 (6)	−0.0024 (5)	−0.0009 (5)	−0.0004 (5)
C1	0.0385 (8)	0.0312 (8)	0.0208 (7)	−0.0012 (6)	0.0023 (6)	0.0016 (5)
C2	0.0252 (7)	0.0295 (7)	0.0222 (7)	−0.0023 (5)	0.0005 (5)	−0.0006 (5)
C3	0.0259 (7)	0.0272 (7)	0.0191 (6)	0.0006 (5)	−0.0022 (5)	−0.0005 (5)
C4	0.0226 (6)	0.0226 (6)	0.0235 (7)	−0.0033 (5)	0.0000 (5)	0.0016 (5)
C5	0.0261 (7)	0.0221 (7)	0.0211 (7)	−0.0030 (5)	0.0002 (5)	0.0020 (5)
C6	0.0323 (7)	0.0271 (7)	0.0259 (7)	0.0009 (6)	0.0039 (6)	0.0032 (5)
C7	0.0434 (9)	0.0311 (7)	0.0256 (7)	−0.0007 (7)	0.0100 (6)	−0.0008 (6)
C8	0.0538 (10)	0.0320 (8)	0.0196 (7)	−0.0073 (7)	0.0006 (6)	0.0016 (6)
C9	0.0397 (8)	0.0306 (7)	0.0258 (7)	−0.0030 (6)	−0.0079 (6)	0.0031 (5)
C10	0.0279 (7)	0.0268 (7)	0.0242 (7)	−0.0008 (5)	−0.0018 (5)	−0.0006 (5)

### Geometric parameters (Å, °)

**Table:**

O—C4	1.2170 (16)	C4—C5	1.4884 (17)
N1—N2	1.3585 (16)	C5—C6	1.398 (2)
N1—C2	1.3351 (18)	C5—C10	1.3998 (19)
N1—C3	1.4476 (16)	C6—H6	0.956 (18)
N2—C1	1.3247 (18)	C7—C6	1.3851 (19)
N3—C1	1.356 (2)	C7—H7	0.954 (18)
N3—C2	1.3258 (18)	C8—C7	1.393 (2)
C1—H1	1.001 (17)	C8—C9	1.386 (2)
C2—H2	0.972 (17)	C8—H8	0.97 (2)
C3—H31	0.974 (18)	C9—H9	0.987 (18)
C3—H32	0.973 (17)	C10—C9	1.390 (2)
C4—C3	1.5195 (18)	C10—H10	1.007 (17)
			
C2—N1—N2	110.05 (11)	C5—C4—C3	116.93 (11)
C2—N1—C3	129.14 (12)	C6—C5—C10	119.22 (13)
N2—N1—C3	120.80 (11)	C6—C5—C4	118.82 (12)
C1—N2—N1	101.81 (11)	C10—C5—C4	121.93 (12)
C2—N3—C1	102.23 (11)	C7—C6—C5	120.26 (14)
N2—C1—N3	115.39 (12)	C7—C6—H6	121.1 (10)
N2—C1—H1	121.1 (10)	C5—C6—H6	118.6 (10)
N3—C1—H1	123.4 (10)	C6—C7—C8	120.28 (14)
N3—C2—N1	110.53 (13)	C6—C7—H7	119.5 (10)
N3—C2—H2	127.4 (9)	C8—C7—H7	120.2 (10)
N1—C2—H2	122.1 (9)	C9—C8—C7	119.79 (13)
N1—C3—C4	112.73 (11)	C9—C8—H8	121.0 (11)
N1—C3—H31	109.1 (9)	C7—C8—H8	119.2 (11)
C4—C3—H31	109.5 (10)	C8—C9—C10	120.31 (14)
N1—C3—H32	109.9 (10)	C8—C9—H9	121.3 (10)
C4—C3—H32	106.3 (10)	C10—C9—H9	118.3 (10)
H31—C3—H32	109.2 (14)	C9—C10—C5	120.11 (13)
O—C4—C5	122.34 (12)	C9—C10—H10	120.4 (9)
O—C4—C3	120.73 (12)	C5—C10—H10	119.5 (9)
			
C2—N1—N2—C1	0.33 (15)	C3—C4—C5—C6	178.95 (12)
C3—N1—N2—C1	−178.72 (12)	O—C4—C5—C10	178.18 (12)
N2—N1—C2—N3	−0.35 (16)	C3—C4—C5—C10	−2.67 (18)
C3—N1—C2—N3	178.60 (12)	C10—C5—C6—C7	−1.1 (2)
C2—N1—C3—C4	93.49 (16)	C4—C5—C6—C7	177.33 (12)
N2—N1—C3—C4	−87.66 (15)	C6—C5—C10—C9	0.3 (2)
N1—N2—C1—N3	−0.21 (17)	C4—C5—C10—C9	−178.08 (12)
C2—N3—C1—N2	0.01 (17)	C8—C7—C6—C5	0.9 (2)
C1—N3—C2—N1	0.20 (15)	C9—C8—C7—C6	0.2 (2)
O—C4—C3—N1	−0.91 (18)	C7—C8—C9—C10	−1.0 (2)
C5—C4—C3—N1	179.92 (11)	C5—C10—C9—C8	0.7 (2)
O—C4—C5—C6	−0.21 (19)		

### Hydrogen-bond geometry (Å, °)

**Table:**

*D*—H···*A*	*D*—H	H···*A*	*D*···*A*	*D*—H···*A*
C2—H2···O^i^	0.970 (16)	2.449 (15)	3.2595 (17)	140.9 (12)
C3—H32···O^i^	0.973 (16)	2.489 (16)	3.2601 (17)	136.1 (13)
C8—H8···N3^ii^	0.97 (2)	2.61 (2)	3.5405 (19)	160.2 (14)
C1—H1···Cg2^iii^	1.001 (17)	2.840 (18)	3.620 (2)	135.20 (13)
C2—H2···Cg1^iv^	0.972 (17)	3.013 (16)	3.829 (2)	142.42 (12)

Symmetry codes: (i) −*x*+1/2, *y*+1/2, *z*; (ii) *x*, −*y*+1/2, *z*−1/2; (iii) −*x*+1, −*y*, −*z*; (iv) −*x*+3/2, *y*+1/2, *z*.
